# *Notes from the Field:* Tetanus in an Unvaccinated Child — Oregon, 2017

**DOI:** 10.15585/mmwr.mm6809a3

**Published:** 2019-03-08

**Authors:** Judith A. Guzman-Cottrill, Christina Lancioni, Carl Eriksson, Yoon-Jae Cho, Juventila Liko

**Affiliations:** ^1^Department of Pediatrics, Oregon Health and Science University, Portland, Oregon; ^2^Public Health Division, Oregon Health Authority.

Tetanus is an acute neuromuscular disease caused by the bacterium *Clostridium tetani*. Bacterial spores found in soil can enter the body through skin disruption, with subsequent onset of clinical illness ranging from 3 to 21 days (usually within 8 days). In 2017, a boy aged 6 years who had received no immunizations sustained a forehead laceration while playing outdoors on a farm; the wound was cleaned and sutured at home. Six days later, he had episodes of crying, jaw clenching, and involuntary upper extremity muscle spasms, followed by arching of the neck and back (opisthotonus) and generalized spasticity. Later that day, at the onset of breathing difficulty, the parents contacted emergency medical services, who air-transported him directly to a tertiary pediatric medical center. The boy subsequently received a diagnosis of tetanus and required approximately 8 weeks of inpatient care, followed by rehabilitation care, before he was able to resume normal activities.

Upon hospital arrival, the child had jaw muscle spasms (trismus). He was alert and requested water but was unable to open his mouth; respiratory distress caused by diaphragmatic and laryngeal spasm necessitated sedation, endotracheal intubation, and mechanical ventilation. Tetanus immune globulin (3,000 units) and diphtheria and tetanus toxoids and acellular pertussis vaccine (DTaP) were administered for presumed tetanus. He was admitted to the pediatric intensive care unit and cared for in a darkened room with ear plugs and minimal stimulation (stimulation increased the intensity of his spasms). Intravenous metronidazole was initiated, and the scalp laceration was irrigated and debrided.

His opisthotonus worsened, and he developed autonomic instability (hypertension, tachycardia, and body temperatures of 97.0°F–104.9°F [36.1°C–40.5°C]). He was treated with multiple continuous intravenous medication infusions to control his pain and blood pressure, and with neuromuscular blockade to manage his muscle spasms. A tracheostomy was placed on hospital day 5 for prolonged ventilator support. Starting on hospital day 35, the patient tolerated a 5-day wean from neuromuscular blockade. On day 44, his ventilator support was discontinued, and he tolerated sips of clear liquids. On day 47, he was transferred to the intermediate care unit. Three days later, he walked 20 feet with assistance. On day 54, his tracheostomy was removed, and 3 days later, he was transferred to a rehabilitation center for 17 days.

The boy required 57 days of inpatient acute care, including 47 days in the intensive care unit. The inpatient charges totaled $811,929 (excluding air transportation, inpatient rehabilitation, and ambulatory follow-up costs). One month after inpatient rehabilitation, he returned to all normal activities, including running and bicycling. Despite extensive review of the risks and benefits of tetanus vaccination by physicians, the family declined the second dose of DTaP and any other recommended immunizations.

This is the first pediatric tetanus case in >30 years in Oregon (unpublished data, Oregon Health Authority, 2018). The diagnosis of tetanus is made based on clinical findings because the bacterium *C. tetani* is difficult to grow from wounds. A wound culture from the child’s laceration did not grow *C. tetani*. However, a negative wound culture does not rule out disease. The health care costs to treat this child’s preventable disease were approximately 72 times the mean (2012) cost of $11,143 for a U.S. pediatric hospitalization (*1*). A recent report describing adult tetanus cases included hospital charges ranging from $22,229 to $1,024,672 (*2*).

Widespread use of tetanus toxoid–containing vaccines (tetanus toxoid inactivated vaccine or a combination vaccine that contains tetanus toxoid) and tetanus immune globulin for wound management has led to a 95% decline in the number of tetanus cases and a 99% decrease in the number of tetanus-related deaths since the 1940s (*3*). From 2009 to 2015, 197 tetanus cases and 16 tetanus-associated deaths were reported in the United States (*4*). Unvaccinated or inadequately vaccinated persons are at risk for tetanus, irrespective of age, and recovery from tetanus disease does not confer immunity (*5*).

Routine administration of a 5-dose DTaP series is recommended for all eligible children at 2, 4, and 6 months of age, then a dose at 15–18 months of age, and a fifth dose at 4–6 years of age. Booster doses of diphtheria and tetanus toxoids are recommended every 10 years throughout life (*4*). Uninsured or underinsured eligible children may receive vaccines at no cost through the Vaccine For Children program (https://www.cdc.gov/vaccines/programs/vfc/index.html). Resources to assist health care providers in discussing vaccination with their patients, including how to address questions, are available online (https://www.cdc.gov/vaccines/partners/childhood/professionals.html).
